# Assessment of perceived risk and precautionary behavior toward COVID-19 pandemic using the health belief model, Saudi Arabia

**DOI:** 10.1186/s42506-022-00111-7

**Published:** 2022-09-21

**Authors:** Eman M. Mortada, Ghada Moh Samir Elhessewi

**Affiliations:** 1 Department of Health Sciences, College of Health & Rehabilitation Sciences, Princess Nourah Bint Abdulrahman University, P.O. Box 84428, Riyadh, 11671 Saudi Arabia; 2 Nursing Administration Department, Faculty of Nursing, Damanhour University, Damanhur, Egypt

**Keywords:** COVID-19 pandemic, Perceived risk, Precautionary behavioral response, Health belief model

## Abstract

**Background:**

The global threat of the COVID-19 pandemic continues to evolve forming the most impactful health crises in modern history, necessities of individuals adhering to mandatory behavior change that limits the spread of the pandemic. The purpose of the current study is to identify behavioral responses of the health sciences university students during the period of the COVID-19 pandemic and determine risk perceptions using the health belief model (HBM).

**Methods:**

A cross-sectional study using an online survey distributed among health sciences female university students in Riyadh, KSA. The questionnaire was used to assess sociodemographic characteristics; knowledge about COVID-19 and its preventive measures, risk perceptions, and beliefs using the HBM; and their actual adoption of precautionary measures.

**Results:**

The mean age of 286 respondents was 21.6 years (SD 2.5). They had good knowledge, positive risk perception, and good practice. Fifty-seven percent of the respondents adhere satisfactorily to COVID-19 precautionary behavior. Respondents with positive overall risk perception had around 6 times significantly higher adherence compared to those with negative risk perception. Perceived benefits have higher odds of adherence to COVID-19 precautionary behavior. Similarly, cues to action were a significant determinant of adherence to COVID-19 precautionary behavior.

**Conclusions:**

The constructs of the HBM provided good measurement of risk perception and the respondent students had good knowledge. Yet, significant gaps were shown between COVID-19 perceived risks and the students’ actual practice of personal hygienic measures, particularly hand hygiene. To put an end to the present COVID-19 and its upcoming waves, it is highly recommended to direct COVID-19 training programs specifically tailored towards university students.

## Introduction

The emergence of highly communicable COVID-19 caused by novel coronavirus that spread globally increasing exponentially creating ever-increasing morbidity and generating alarming mortality rates created a global emergency situation and caused an outburst of health systems in many countries worldwide [1]. In response to this, the World Health Organization (WHO) affirmed COVID-19 as a public health emergency of international concern on the 30 January 2020 and called for collaborative efforts of all countries to prevent the rapid spread of COVID-19 producing one of the largest global health crises in modern history [2]. The WHO has officially declared on March 11, 2020, that COVID-19 is a global pandemic, after remarkable proliferation overcoming geographical barriers and sweeping more than 210 countries and territories [3].

The Kingdom of Saudi Arabia (KSA) has been severely hit by this fatal virus, so KSA has to combat the unprecedented hike in a number of reported COVID-19 positive cases recording the largest number of infections in a single Arab country, with a number of confirmed cases are continuing to evolve [4, 5]. A rapidly escalating COVID-19 pandemic could have a detrimental effect on the psychological health, economic, social, and religious aspects of life and has created a massive threat to humanity that could potentially have long-term profound impacts on public health. In response to this, governments recognize the importance of applying precautionary measures to restrain the disease. Public behaviors and population co-operation are extremely vital to curb the spread of infection and to put an end to the pandemic. The engagement to preventive behaviors is the most effective option to overcome the disease and is highly recommended to halt the spread of COVID-19. Consequently, the WHO advised the general public to follow the precautionary measures that generally fell into one of the two following categories: preventive measures (e.g., wearing a mask, washing hands, using a hand sanitizer for maintaining hand hygiene) and social distancing behaviors (e.g., reducing the use of public transport, avoiding crowded places and postponing or canceling social events and staying at home) [6, 7].

Similarly, on February 1, 2020, the KSA started executing strict precautionary measures such as suspending all flights from China. Afterwards, the kingdom banned all incoming and outgoing flights. On Monday, March 9, 2020, all educational systems in KSA were temporarily suspended and replaced by distant on-line learning using virtual classes. This was followed by a nationwide curfew under which exiting homes is only permissible for adults during specific times. Most of the hospitals have also implemented limited exposure to patients, visitors, and staff and thus postponed nonurgent surgeries and visits to outpatient clinics [8].

Implementation of non-pharmaceutical public health interventions should be based on the understanding of the public’s perceptions and beliefs towards health threat that may be necessary for determining adherence with health advice [9]. The public may be more likely to adhere with health recommendations if they believe that they are effective [10]. For behavior change to successfully occur, people must see the coronavirus as a threat to their health, understand that adopting the new behavior (physical distancing) will result in positive outcomes/benefits, and feel confident in their ability to overcome any perceived barriers that may get in their way of taking action.

**Fig. 1:**
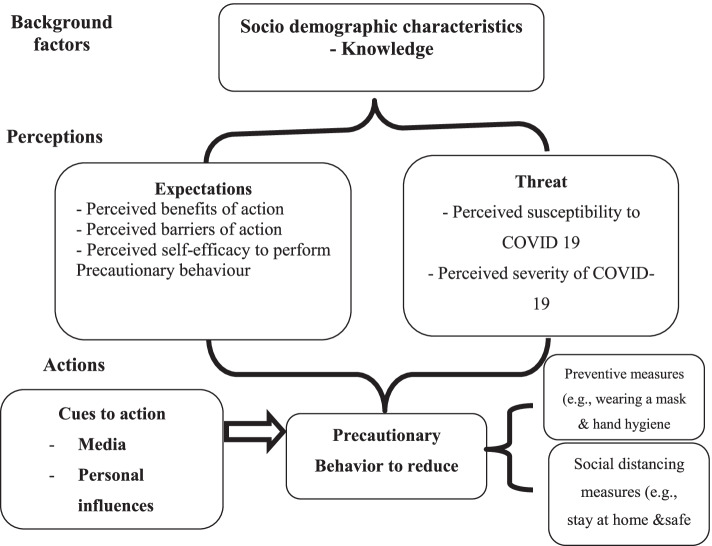
Conceptual framework of adapted HBM and its main constructs [13, 14]

The health belief model (HBM) is the most commonly proposed model to explain the factors influencing the health behavior and is used as a conceptual framework and provide a theoretical guideline for health behaviors in the public health research, Thus, the model can be helpful in explaining adoption of preventive health behaviors in society is essential to better control the (COVID-19) pandemic by understanding individual’s beliefs and attitudes [11–14]. Conceptually, HBM is composed of six basic constructs, which include perceived susceptibility, perceived severity, perceived benefits, perceived barriers, perceived self-efficacy which refers to level of self-confidence, and cues to action which encourage people to implement preventive health behaviors [13, 14] as shown in Fig. 1.

All these constructs are influenced by an individual’s background, and the risk perception of being infected by the coronavirus is mediated by the type of information that the individual hold; these factors untimely could influence the emotional and behavioral reactions towards COVID-19 and can explain the individual decision to adopt preventive health. Consequently, their risk perceptions and behaviors are more affected by this pandemic [15, 16]. The objectives of the present study are to identify the precautionary behavioral responses of female Saudi university students enrolled in the faculties of health sciences during the period of the COVID-19 pandemic, identify factors that contribute to their behavioral response, and determine their risk perceptions based on the HBM.

## Methods

### Study design and setting

A cross-sectional study was conducted in *Princess Nourah bint* Abdulrahman University (PNU) in Riyadh, KSA, after the lockdown period of the COVID-19 pandemic for 2 months period from June to July 2020.

### Target population

Female university students are the target for the study, being in Saudi, enrolled in any of the 5 health sciences colleges at PNU, and agreed to participate in the study which were the inclusion criteria.

### Sample size and sampling technique

A snowball and convenient sampling technique were used to ensure maximal participation, whereas participants were asked to share the survey with their social media networks. The required sample size is 286 for the study calculated using OpenEpi to give 80% degree of precision at 95% confidence interval (CI) and 70% rate of adopting precautionary behavior from a previous study [17].

### Data collection

#### The tool

A structured online questionnaire designed on Google forms was used to collect required data measuring the dependent variable (adhering to precautionary behavior practiced against COVID-19) and the independent variables including demographic characteristics, level of knowledge, and risk perception using six constructs of HBM. The questionnaire consisted of several questions categorized into four sections:

*The first section* covers sociodemographic characteristics: including age, marital status, having children, and monthly income. *The second section* includes items assessing the level of knowledge about COVID-19 infection [18] and comprised of 12 items: knowledge about modes of infection with COVID-19 (three items), manifestations that patients suffer from (two items), high-risk group (1-item), and levels of its prevention and control (six items). Response to each question is either yes or no and scored 1 and 0 for correct answer and incorrect answer respectively. The total knowledge score was calculated; it ranged from 0 to 12; the results were dichotomized by using the median as a cut-off point with those who have scores equal or above the median were classified as having good knowledge level and those who had a score below the median were classified as having poor knowledge [19]. *The third section* includes questions adapted from the tailored version to assess perceptions toward COVID-19 through using 6 constructs of HBM [17, 18] two items assessing perceived susceptibility that refers to individual’s belief regarding the chances of contracting COVID 19 infection, seven items assessing perceived severity which is person’s belief of how serious the virus is alongside with the probable consequences of being infected with COVID 19, eight items perceived barriers cons of adopting the new preventive health behavior, three items assessing perceived benefits that pros of adopting the new preventive health behavior, and cues to action (one item). The items were rated on a 5-point scale, ranging from “strongly disagree = 1” to “strongly agree = 5.” In addition to three items measuring perceived self-efficacy which refers to the level of self-confidence and perceived ability to exert personal control in being engaged in a particular behavior (scored on a 5-point scale, ranging from “unsure = 1” to “very sure = 5”). A total score was calculated by adding up the responses of respective Likert scale for item scores in each construct. Then, the results were dichotomized by using the median as a cut-off point into high perceived construct (scores equal or above the median) and low perceived construct (scores below the median). A composite score for COVID-19 risk perception was calculated by adding up the scores of the HBM constructs then subtracting the score for perceived barriers from that for perceived benefits, to create a net benefits score. Then, the result was dichotomized by using the median as a cut-off point into positive risk perception (scores equal or above the median) and negative perceived risk (scores below median) [19].

*The last section* is composed of seven items, assessing the level of adherence to COVID-19 precautionary behavior [20, 21] that includes two different categories: hygienic measures such as wearing masks, practicing proper hand hygiene, and social distancing behaviors such as avoiding crowded places and staying at home. Seven questions were used for asking the respondents how frequently they were adhering to these precautionary behaviors using a 5-point scale, ranging from “never = 1” to “always = 5,” with the total score ranging from 7 to 35 after summing the responses. Accordingly, the health sciences university students were classified into those with satisfactory level of adherence (scores above the median) and unsatisfactory level of adherence (scores equal or below median) [19].

#### Data collection procedure

The link was shared through online platforms on social media such as Twitter, Telegram, and WhatsApp groups after being pilot tested on a sample of 10 persons to assess the feasibility, the comprehension of the questionnaire items, and the time required for data collection and to assess internal consistency of different questionnaire items. This pilot tested responses was excluded from the final analysis and required modifications made accordingly. Then, reliability testing was conducted for the total knowledge, risk perception, and adherence to COVID-19 precautionary behavior using Cronbach’s alpha coefficients which were.73, .79, and .69, respectively, indicating that the tool is reliable.

### Statistical analysis

Data was analyzed using the Statistical Package for the Social Sciences (SPSS) version 20.0 (SPSS, Chicago, IL, USA). Internal consistency was assessed by Cronbach alpha coefficients (*α*). Descriptive analysis was performed by mean, standard deviations for quantitative data and frequencies, and percentages for categorical variables as applicable. Chi-square test was used to measure the associations between the dependent variable (adhering to precautionary behavior practiced against COVID-19) and independent variables including demographic characteristics, level of knowledge, and risk perception using six constructs of HBM. Logistic regression analysis was performed to predict potentially significant determinants of behavior, including all variables that were significantly associated with the dependent variables in the bivariate analysis. *P*-value of ≤ .05 was considered statistically significant. Spearman correlation test was used to find correlation between knowledge, attitude, and practice sections.

## Results

**Fig. 2:**
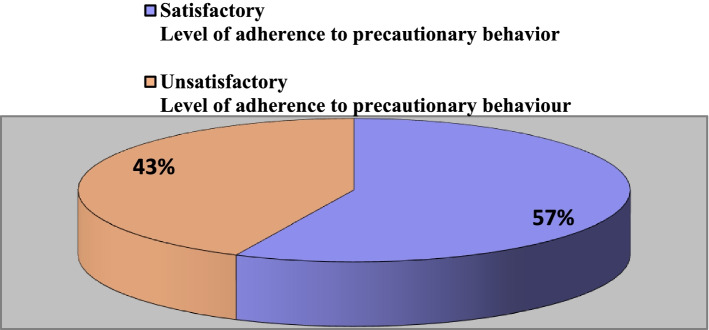
Level of adherence to COVID-19 precautionary behavior among the students of health sciences in Princess Nourah University, 2020 (*n* = 286)

Three hundred and eleven study participants responded to the online questionnaire. After excluding 25 ineligible and incomplete responses, the final sample consisted of 286 respondents (Fig. 2). Overall, the mean age of respondents was 21.6 ± 2.5 years. Among them, 48.3% were from the health science and rehabilitation college, 14.3% medicine, 9.4% dentistry, 6.3% pharmacy, and 21.7% were nursing students. Around 46% of the students just finished their second year in their respective colleges. In the majority of the students (71.0%), their family earning was more than 10,000 Saudi Riyal monthly. A minority of the respondent students were married (12.2%), and only half of these were having children (6.6%).

The behavioral responses of the sampled students to seven items of precautionary behaviors assessing the threat of COVID-19 are illustrated in Table 1. The first most prevalent behaviors as reported by the vast majority of students (80.8%) were always wearing masks when going outside the home followed by always maintaining safe distance (at least one meter) from others when going outside the home (62.6%). Then comes in the 3rd place (51.0%) both always wearing rubber gloves in public and staying at home and do not leave the house unless absolutely necessary. Meanwhile, only18.9% reported that they always wash their hands regularly with soap and water for at least 20 s every hour. The mean score of COVID-19 precautionary behavior was 27.71 ± 4.57 ranging from 7 to 35, 79.1% (27.7/35*100), indicating good overall behavior.

**Table 1:** Responses to COVID-19 precautionary behaviors among the students of health sciences in Princess Nourah University, 2020 (*n* = 286)

Categories of precautionary behaviors	Never No. (%)	Seldom No. (%)	Sometime No. (%)	Often No. (%)	Always No. (%)
**Social distancing measures**					
o Stay at home	11 (3.8)	2 (0.7)	39 (13.6)	88 (30.8)	146 (51.1)
o Maintain safe distance	2 (0.7)	8 (2.8)	19 (6.6)	78 (27.3)	179 (62.6)
**Personal hygienic measures**					
o Wearing masks when going out	10 (3.5)	3 (1.0)	15 (5.2)	27 (9.4)	231 (80.8)
o Wearing rubber gloves in public	43 (15.0)	18 (6.3)	36 (12.6)	43 (15.0)	146 (51.1)
o Wash my hands as recommended	21 (7.3)	44 (15.4)	100 (35.0)	67 (23.4)	54 (18.9)
o Do not touch eyes, nose, and mouth by hands	22 (7.7)	36 (12.6)	95 (33.2)	84 (29.4)	49 (17.1)
o Disinfecting touchable things	12 (4.2)	27 (9.4)	60 (21.0)	71 (24.8)	116 (40.6)
**Precautionary behaviors**					
o **Total score(*X*** **± SD)**	27.7 ± 4.5				
o **%**	27.7/35*100 = 79.1				

According to findings from Fig. 2, one hundred and sixty-three (57.0%) of the students satisfactorily adhered to precautionary behavior against COVID 19.

A statistically significant association is noticed in those having children and the year they finished in their colleges (*P* ≤ .05) where satisfactory adherence to COVID-19 precautionary behavior was higher among those who just finished 4th year (58.1%), compared to remaining years. Similarly, adherence was more than 7 times higher among students not having children (OR: 7.97, 95% CI: 2.26–28.77, *P* <.001). However, there is no statistically significant differences in the level of adherence between students from different age groups and those who are married or not (Table 2).

**Table 2:** Adherence to COVID-19 precautionary health behavior according to sociodemographic characteristics of the students of health sciences university in Princess Nourah University, 2020 (*n* = 286)

Characteristics	Study groups		Total No. (%)	*𝛘*^2^ test	***P*** value	OR (95%CI)
	Level of adherence to precautionary behavior					
	Satisfactory No. (%)	Unsatisfactory No. (%)				
**Age groups(years)**						
≤ 20	68 (61.8)	42 (38.2)	110 (38.5)	2.89	.24	1
21–22	57 (57.6)	42 (42.4)	99 (34.6)			1.19(0.66-2.16)
≥ 23	38 (49.4)	39 (50.6)	77 (26.9)			1.66(0.88-3.13)
χ ± SD	21.6±2.5					
**Year finished in college**						
1st year	27 (77.1)	8 (22.9)	35 (12.2)	10.1 ^**a**^	.018*	1
2nd year	73 (55.3)	58 (44.7)	132 (46.2)			0.72 (0.39–1.33)
3rd year	45 (59.2)	31 (40.8)	76 (26.6)			0.77 (0.39–1.50)
4th year	118 (41.9)	25 (58.1)	43 (15.0)			0.59 (0.24–1.22)
**Type of college**						
CHSR	78 (56.5)	60 (43.5)	138 (48.3)	4.54 ^**a**^	.324	1
Medicine	24 (58.5)	17 (41.5)	41 (14.3)			2.22 (1.16–4.24)
Density	18 (66.7)	9 (33.3)	27 (9.4)			0.65 (0.25–1.66)
Pharmacy	13 (72.2)	5 (27.8)	18 (6.3)			0.50 (0.15–1.62)
Nursing	30 (48.4)	32 (51.6)	62 (21.7)			1.39 (0.73–2.64)
**Marital state**						
Married	18 (57.8)	17 (42.2)	35 (12.2)	0.51	.59	1
Not married	145 (51.4)	106 (48.6)	251 (87.8)			1.29 (0.64–2.62)
**Having children**						
No	160 (59.9)	107 (40.1)	267 (93.4)	14.05	< .001*	1
Yes	3 (15.8)	16 (84.2)	21 (6.6)			7.97 (2.26–28.77)
**Family income (**SR ):						
< 10,000	46 (55.4)	37 (44.6)	83 (29.0)	3.66	.16	1
10,000–20,000	66 (64.1)	37 (35.9)	103 (36.0)			0.70 (0.37–1.31)
> 20,000	51 (51.0)	49 (49.0)	100 (35.0)			1.19 (0.64–2.24)
**Total**	163 (57.0)	123 (43.0)	286 (100.0)			

*P ≤ 0.05 is significance

^a^calculated using fisher test

*OR* Odds Ratio, *CI* Confidence Interval, *CHSR* College of Health and Rehabilitation Sciences

Table 3 displays the comparison between levels of adherence to COVID-19 precautionary behavior and perceived risks using six constructs of HBM. Despite, students who perceived high susceptibility and severity (59.8 and 58.4%) showed better adherence, yet there is no significant association that exists between them and those with perceived low susceptibility and severity (*P* >.05). A statistically significant association was observed between students’ level of adherence and their perceived benefits and perceived barriers, self-efficacy, and cues of action (*P* <. 001). Students who perceived their benefits to be high had around 2 times significantly higher adherence compared to those who perceived low benefits (OR: 2.43, 95% CI: 1.44–4.1, *P* <.001). Respondent students with high-perceived barriers (56%) are less likely to satisfactorily adhere to COVID-19 precautionary behavior compared to those with low perceived barriers. (59.5%) (*P* <.001). Those with high perceived cues to action and high self-efficacy had around 4 and 3 times more satisfactorily adherent than those with low perceived cues to action and self-efficacy (OR = 3.89, CI 2.31–6.58 and OR = 2.7, CI 1.7-4.5 respectively) (*P* <.001), similarly.

**Table 3:** Risk perception using 6 construct of health beliefs among health sciences university students according to their level of adherence to COVID-19 precautionary behavior (*n* = 286)

Characteristics	Study groups		Total No. (%)	*𝛘*^2^ test	***P*** value	0R (95%CI)
	Level of adherence to precautionary behavioral					
	Satisfactory No. (%)	Unsatisfactory No. (%)				
**Perceived susceptibility**						
High	104 (59.8)	70 (40.2)	174 (60.8)	1.39	.237	1
Low	59 (52.7)	53 (47.3)	112 (39.2)			1.34 (0.88–2.15
**Perceived severity**						
High	87 (58.4)	62 (41.6)	149 (52.1)	0.25	.62	1
Low	76 (55.5)	61 (44.5)	137 (47.9)			1.13 (0.71–1.80)
**Perceived benefits**						
High	129 (63.2)	75 (36.8)	204 (71.3)	11.3	<.001*	1
Low	34 (41.5)	48 (58.5)	82 (28.7)			2.43 (1.44–4.1)
**Perceived barriers**						
High	66 (44.0)	84 (56.0)	150 (52.4)	21.7	<.001*	1
Low	97 (71.3)	39 (28.7)	136 (47.6)			0.31 (0.19–0.52)
**Perceived self-efficacy**						
High	123 (65.4)	65 (34.6)	188 (65.7)	15.9	<.001*	1
Low	40 (40.8)	58 (59.2)	98 (34.3)			2.7 (1.7–4.5)
**Cues to action**						
High	131 (67.5)	63 (32.5)	194 (67.8)	27.3	<.001*	1
Low	32 (34.8)	60 (65.2)	92 (32.2)			3.89 (2.31–6.58)
**Risk perception**						
Positive	119 (75.3)	39 (24.7)	158 (55.2)	48.4	<.001*	1
Negative	44 (34.4)	84 (65.6)	128 (44.8)			5.83 (3.49–9.74)
**COVID-19-related knowledge**						
Good	105 (59.0)	73 (41.0)	178 (62.2)	0.77	.38	1
Poor	58 (53.7)	50 (46.3)	108 (37.8)			1.24 (0.77–2.1)

*P ≤ 0.05 is significance

*OR* Odds Ratio, *CI* Confidence Interval

Despite no significant association existing between the students’ level of knowledge about COVID-19 and their level of adherence to precautionary behavior (*P* >0.05), those with good knowledge (59.0%) showed better adherence. On the other hand, students with positive overall risk perception had around 6 times significantly higher adherence compared to those with negative risk perception (OR: 5.83, 95%CI: 3.49–9.74, *P* <.001) (Table 3).

The results of the Spearman correlation coefficient revealed a moderately significant correlation between COVID-19 precautionary behavior and risk perception (*r* = 0. 0.54, *p* < .001) in addition to a weak significant correlation between knowledge and both risk perceptions and adherence to precautionary behavior (*r* = 0.26 and 0.19, *P* < 0.001, respectively). Regarding the six constructs of the HBM, all were positively correlated with the adherence to precautionary behavior except perceived barriers which is negatively correlated with COVID-19 precautionary behavior, risk perception, and level of knowledge (*r* = − 0.52, − 0.77, and − 0.23, *P* <.001, respectively) (Table 4).

**Table 4:** Correlation between COVID-19 precautionary preventive behavior, related knowledge, and constructs of the health belief model among health sciences students

Variables	Correlation results for the variables								
	1	2	3	4	5	6	7	8	9
1. **Precautionary behavior**	1								
2. **Risk perception**	0.54^b^	1							
3. **Knowledge**	0.26^b^	0.19^b^	1						
4. **Perceived susceptibility**	0.07	0.29^b^	− 0.07	1					
5. **Perceived severity**	0.005	0.39	− 0.16^b^	0.12	1				
6. **Perceived barriers**	− 0.52^b^	− 0.77^b^	− 0.23^b^	0.12^a^	0.13^a^	1			
7. **Perceived benefits**	0.25^b^	0.42^b^	0.16^b^	0.06	0.09	− 0.14	1		
8. **Perceived self-efficacy**	0.41^b^	0.58^b^	0.28^b^	− 0.40	− 0.33	0.43^b^	0.19^b^	1	
9. **Cues to action**	0.31^b^	0.46^b^	0.12^a^	0.08	0.02	− 0.27^b^	0.23^b^	0.34^b^	1

^a^Correlation is significant at 0.05 level

^b^Correlation is significant at 0.01 level

Binary logistic regression analysis reveals that the factors that significantly predict COVID-19 precautionary protective behavior among the female university students are both perceived benefits and cues to action (*P* < .05) as perceived benefits (OR: 1.85, 95% CI: 1.03–3.32, *P* = .038) have higher odds of adherence to COVID-19 precautionary protective behavior. Similarly, cues to action (OR: 2.060, 95% CI: 1.13–3.76, *P* = .018) was also a significant predictor of adherence to COVID-19 precautionary behavior (Table 5).

**Table 5:** Logistic regression for factors predicting adherence to COVID-19 precautionary behavior in health sciences female university students, KSA, June–July 2020

Variable	B	Wald	***P*** value	Β	95% C.I. for β	
					Lower	Upper
**Knowledge level**	.001	.000	.999	1.001	.575	1.742
**Perceived susceptibility**	− .069	.060	.807	.93	.536	1.625
**Perceived severity**	− .018	.004	.949	.98	.559	1.725
**Perceived barriers**	− .264	.576	.448	.768	.389	1.518
**Perceived benefits**	.617	4.298	.038*	1.85	1.034	3.320
**Perceived self-efficacy**	.291	.887	.346	1.34	.730	2.451
**Perceived cues to action**	.723	5.560	.018*	2.060	1.130	3.756

*P ≤ 0.05 is significance, *B* unstandardized beta “regression coefficient,” *β* standardized beta

## Discussion

This study provided clear mapping of the perceived risk and precautionary behaviors towards COVID-19 infection risk through the use of the HBM. Female university students were the target of the study as their positive attitudes towards adopting precautionary behavior of those young adults can be promoted to combat COVID-19; consequently, they will play an important role in raising awareness of the Saudi community. Regarding precautionary behavior, most of the respondents (80.8%) reported that they were always wearing masks when going outside the home and were always maintaining a safe distance (62.6%). Additionally, 51.0% reported both always wearing rubber gloves in public and staying at home and do not leave the house unless absolutely necessary.

These results are consistent with a recent study done in a Chinese population where nearly all of the participants (98.0%) admitted to wearing masks when leaving their homes [16] and another study done on medical students in Jordan where 83.1% reported staying at home. However, [21] these results are in contrast to the study in Jordan where only 9.7% of medical students considered wearing a face mask often. This could be due to the educational level of the university sampled students and the strict government control measures and regulations enforced in KSA [22]. Furthermore, such face mask practices are advised by the WHO and a previous study [23, 24]. Better education regarding the need for regular hand washing with soap and water for at least 20 s every hour is essential as these practices are also advised by the WHO. COVID-19 precautionary behavior among health sciences university students indicates good overall behavior. This result is in accordance with a study carried out on the Vietnamese population which indicates excellent preventive behavior [25]. This may be due to the relatively rapid and serious regulations established by government to reduce the spread of COVID-19 infection in KSA, in addition to the unprecedented working methods applied by the universities and most of the organizations in accordance with national efforts to promote working from home.

A statistically significant association is noticed in those having children and the year they just finished in their colleges. This result is alongside with the results of a survey done in Vietnam which revealed that young adults were moderately to severely be worried or afraid about the health risks for their family members, and this was more than the level of concern about their own health [25].

The result of this study revealed that health sciences university female students with good knowledge showed better adherence than those with low knowledge level. This result has been supported by another study in Bangladesh which found that females with higher education and more knowledge about COVID-19 displayed more precautionary behaviors than their counterparts [24].

Students who had high perceived risk of getting COVID-19 had around 6 times significantly higher adherence to the precautionary behaviors compared to those with low risk perception. This result is probably because the study sample is highly educated health sciences students and can understand the severity of the outbreak in their community. Moreover, this relation can be explained by our sample student’s belief that the disease is a serious one. An opposite result was reported in a study carried out in Iran targeted final year medical students [26]; this study showed a negative correlation between preventive behavior and risk perception.

The mean knowledge score was overall 88.6% reflecting good knowledge level. This is expected as health sciences university students have demonstrated more advanced knowledge related to health care issues; besides, they are commonly referred to for healthcare advice from family and friends [27, 28]. Similarly, the mean risk perception score towards developing COVID-19 indicated positive risk perception. This may be due to the widely disseminated information via mainstream and social media about outbreaks in healthcare, religious gatherings, and entertainment facilities. This is in accordance with a similar study in Turkish university healthcare students where social media was a major information source for learning about the influenza pandemic [28].

Regarding the six constructs of the HBM, all were directly correlated with the adherence to precautionary behavior. This is consistent with the evidence-based HBM which theorizes that the ability to follow every preventive instruction against COVID 19 infection and willingness to take action and adhere to precautionary behavior is inspired by individuals’ beliefs about whether they are at risk for health problem or not, and how they perceive the benefits of taking action to avoid this problem explains and predicts modifications of individuals’ health-related precautionary behaviors to lessen threat to health [30].

What is noteworthy to find is that more than half of respondents (63.8 and 53.4%) who perceived high susceptibility and severity showed better adherence to COVID-19 precautionary behavior. This result goes alongside with a study carried out in Iraqi Kurdistan Region which revealed that individuals believed that the disease is a serious one, and they were adherent to the preventive measures [31]. This result is expected as the perception of greater symptom severity may lead people to adhere to precautionary behaviors earlier. Besides, the perceived susceptibility and severity modify behaviors in a way that an individual is more likely to take precautionary healthy behaviors seriously if the consequences of getting infected by COVID-19 (e.g., hospitalization, pneumonia, and death) is great.

Another important result which was obtained as a statistically significant association was observed between students’ level of adherence and their perceived benefits and perceived barriers, self-efficacy, and cues of action. This result is expected and supported by the HBM which suggests that individual perceptions and direct cues to action inform behavior. In our study, students’ perceptions are informed by their medical background and influenced by the messages made through news and media reports and government policy actions throughout the course of the outbreak. These messages in turn changed their behaviors through increased adherence to the COVID-19 prevention behaviors as it targeted their perceived benefits, perceived barriers, and ability to take action (i.e., perceived self-efficacy) to reduce transmission of COVID-19. Those with high perceived cues to action and high self-efficacy had around 4 and 3 times more satisfactorily adherence than those with low perceived cues to action and self-efficacy. Perceived self-efficacy is about the ability to follow every preventive instruction against COVID-19 infection by reinforcing positive steps and the belief that one has ability to overcome a given situation. Moreover, effective COVID-19 mitigating behaviors require significant efforts to strengthen beliefs about the disease which includes the severity and susceptibility of threat through TV and radio information about COVID-19.

Findings of this study revealed that both perceived benefits and cues to action significantly were determinants of COVID-19 precautionary protective behavior adherence among female university students. The results propose that TV and radio information about COVID-19 has been helpful ways for interventions and informing individuals about the efficacy of wearing masks, hand washing, social distancing, and staying at home and perhaps also elevating the importance of general health precautionary protective behavior and voluntary compliance with government rules and recommendations [17]. On the other hand, students with high-perceived barriers (40.5%) were less prone to adhere to COVID-19 precautionary behavior compared to those with low perceived barriers. (59.5%). This result is predictable due to the considerably increased difficulties, challenges, and negative effects of physical distancing (e.g., forbidden family gatherings, loss of freedom, violation of individual rights, inconvenience, loss of income, etc.) as well as perceived uselessness of physical distancing.

### Limitations of the study

The results of the study cannot be generalized as the circumstances of COVID-19 and its lockdown measures enforced the researchers to use nonprobability sampling technique, in addition to social desirability bias with high tendency to report positive findings.

## Conclusions

Overall, health sciences students at the Princess Nourah bint Abdulrahman University in Saudi Arabia reported good adherence to precautionary measures and showed good levels of risk perception regarding COVID-19. Countries where the epidemic is hitting hard should implement strategies to keep their university students updated about emerging public health and medical emergencies. To that end, policy makers should be alert to the importance of the HBM’s constructs which can be employed to facilitate COVID-19 precautionary health behaviors. Furthermore, future research is needed to determine whether our findings are generalizable to broader students’ population.

## Data Availability

Datasets used in the current study are available from the corresponding author on reasonable request.

## Abbreviations

HBM: Health belief model
KSA: Kingdom of Saudi Arabia
PNU: Princess Nourah bint Abdulrahman
CHSR: College of Health and Rehabilitation Sciences
